# Needle aspiration for management of acute intraoperative fluid misdirection during phacoemulsification

**DOI:** 10.1016/j.ajoc.2022.101773

**Published:** 2022-12-09

**Authors:** Ninel Z. Gregori, Patrick Staropoli, Swarup Swaminathan, Ryan J. Zukerman

**Affiliations:** a Department of Ophthalmology, Bascom Palmer Eye Institute, University of Miami Miller School of Medicine, 900 NW 17th Street, Miami, FL, USA; b Miami Veterans Affairs Medical Center, 1201 NW 16th Street, Miami, FL, USA; c Department of Ophthalmology, Edward S. Harkness Eye Institute, Columbia University Irving Medical Center, New York-Presbyterian Hospital, New York, NY, USA

## Abstract

**Purpose:**

Acute intraoperative fluid misdirection is a serious complication that may occur during phacoemulsification. We provide a detailed description and a video of our preferred technique for prompt management of acute intraoperative fluid misdirection.

**Observations:**

A 79-year-old male developed sudden shallowing of the anterior chamber and marked elevation of intraocular pressure at hydrodissection during phacoemulsification surgery. Treatment consisted of a needle aspiration of trapped fluid from the retrolental space, employing a 5/8th inch, 25-gauge needle on a medium-size syringe leading to immediate softening of the globe and deepening of the anterior chamber. The rest of the case proceeded uneventfully. The patient had uncomplicated recovery and final best-corrected visual acuity of 20/20.

**Conclusions and importance:**

Acute subcapsular infusion fluid entrapment may occur during uneventful phacoemulsification. Needle aspiration of retrolental fluid is a simple and inexpensive method for immediate resolution of high IOP and deepening of the anterior chamber, allowing the case to proceed.

## 1 Introduction

The phenomenon of acute intraoperative misdirection of infusion fluid into the posterior segment leading to marked posterior pressure is a rare but potentially serious complication during anterior segment surgery.^1^^,^^2^ Subcapsular infusion fluid entrapment, or misdirection, usually occurs during uneventful phacoemulsification. The surgeon notices a sudden rock-hard eye associated with anterior displacement of the iris-lens diaphragm and a centrally and peripherally shallow anterior chamber in the absence of retrobulbar hemorrhage or suprachoroidal hemorrhage or effusion. Irrigation forces balanced salt solution or viscoelastic through the zonules into the posterior chamber and then into either the retrolenticular Berger's space or the anterior vitreous.^1^ It can occur during hydrodissection, irrigation/aspiration, or toward the end of phacoemulsification when the posterior chamber-anterior hyaloid membrane barrier may be disrupted.^2^ It may also occur if large amounts of viscoelastic are injected into the sulcus for a sulcus lens implantation or endocyclophotocoagulation. Here we describe our preferred needle aspiration technique, which has been successful in managing such cases.

## 2 Case report

**Fig. 1:**
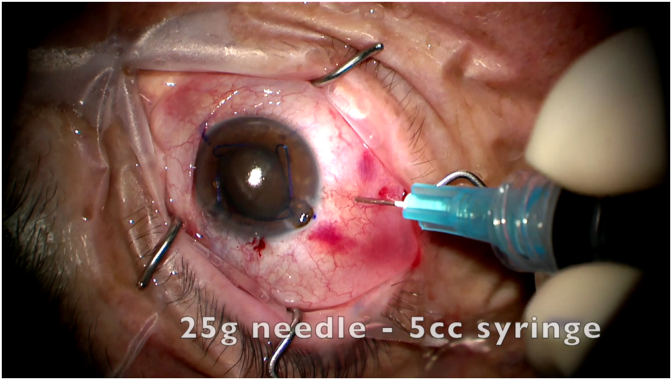
Figure shows iris prolapse into the main phacoemulsification wound due to retrolental balanced salt accumulation during hydrodissection leading to very high intraocular pressure. A 5/8^th^ inch 25-gauge needle on a 5-milliliter syringe is inserted through the conjunctiva at a distance of 4.0 millimeters (mm) posterior to the corneal limbus in this phakic eye.

**Fig. 2:**
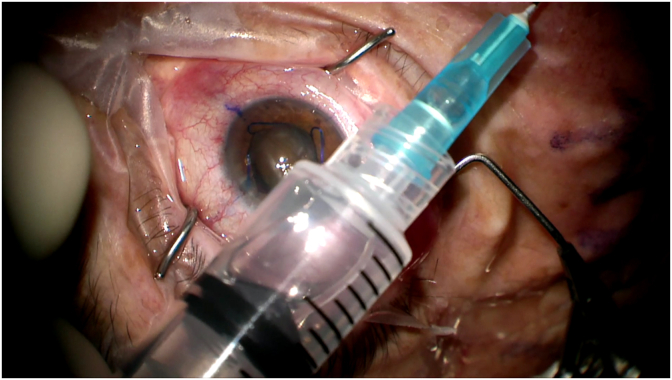
Figure shows balanced salt solution was aspirated into the syringe and the eye immediately softened.

A 79-year-old male presented for cataract extraction with a very dense crystalline lens and a small pupil. Preoperative best-corrected visual acuity was 20/100. Topical anesthesia was applied. Trypan blue and Malyugin ring were utilized. During hydrodissection the anterior chamber was noted to suddenly shallow, and the eye became rock-hard. Efforts to express viscoelastic through the corneal wounds failed to soften the eye, and the iris was seen protruding into the main wound. Thus, a small amount of subconjunctival lidocaine was administered for patient comfort, and then a 5/8th inch 25-gauge needle on a 5-mL syringe was inserted through the conjunctiva at a distance of 4.0 mm posterior to the corneal limbus (Fig. 1). The needle was inserted perpendicularly to the eye wall without a bevel, half-way to three-quarters-way into the eye. Next, the plunger was pulled back to initiate aspiration of the misdirected irrigation solution. Initially no fluid entered the syringe resulting in a “dry tap”. Then, the tip of the needle was moved side to side and tilted slightly anteriorly toward the capsule while pulling back on the plunger to search for the fluid pocket (supplemental video 1). Balanced salt solution was then seen to enter the syringe, and the eye immediately softened. At that point we stopped the aspiration and withdrew the needle (Fig. 2). Approximately 0.5 mL were removed. The anteriorly chamber was reformed with viscoelastic, and the iris was reposited from the wound. The nucleus and the cortex were then removed in a routine fashion without reaccumulation of misdirected fluid. A toric intraocular lens was implanted, and Malyugin ring removed. The patient had a routine postoperative course and a final best-corrected visual acuity of 20/20.

Supplementary video related to this article can be found at https://doi.org/10.1016/j.ajoc.2022.101773.

The following is the supplementary data related to this article:

Supplemental Video 1 Video demonstrates our needle aspiration technique for management of acute intraoperative fluid misdirection. Subconjunctival lidocaine, needle insertion, initial “dry tap”, needle reposition, and fluid aspiration are demonstrated. Supplemental Video 1

## 3 Discussion

The optimal treatment for acute irrigation fluid misdirection syndrome has been controversial. Liberation of retrolenticular fluid via a needle aspiration, one-port vitrectomy with utilization of a single small-gauge (23-, 25-, or 27- gauge) trocar/cannula for vitrector insertion, and even a full three-port vitrectomy have all been described.^1^, ^2^, ^3^, ^4^, ^5^ In their recent article, Grzybowski and Kanclerz reviewed the current studies on the subject, and suggested that it would be preferable to use a small-gauge (23-, 25-, or 27- gauge) trocar/cannula and a vitrector rather than a needle to achieve decompression due to concerns of vitreous traction and inconsistent results with needle aspiration.^2^

Over the past 17 years in practice, we encountered approximately 6 cases of acute intraoperative fluid misdirection and successfully managed all by using a short 5/8th inch or 1-inch 25-gauge needle as described here. Since the iris-lens diaphragm is displaced anteriorly in these cases, and the needle enters the eye perpendicularly at 4.0 mm (if still phakic) or 3.5 mm (if pseudophakic) posterior to the limbus, the likelihood of injuring the lens or the posterior capsule is minimized. The technique is similar to a vitreous tap used for obtaining vitreous cultures. Typically, balanced salt solution will be seen to enter the syringe immediately or after repositioning the needle as shown in the video, and the eye will immediately soften. The rest of the case typically proceeds in a routine fashion without reaccumulation of misdirected fluid.

We highly prefer a simple needle aspiration to utilizing a vitrector in these cases for several reasons. Firstly, the IOP can be immediately normalized since no additional instrumentation or machine set up is required, minimizing retinal and optic nerve ischemia in the setting of prolonged high IOP. Secondly, a needle aspiration carries a much smaller risk of inadvertently cutting the posterior capsule compared to a cutter. Additionally, a needle exerts far less traction on the vitreous base than a transconjunctival cannula and vitreous cutter. Since a retinal examination with scleral indentation may be difficult with a hazy cornea at the end of phacoemulsification, potential retinal breaks caused by the cannula insertion and the vitreous cutter, which are not uncommonly seen in vitreoretinal procedures, could result in a higher risk of retinal detachment. Finally, avoiding the need for opening additional instrumentation, seeking the assistance of a retinal surgeon to help with the vitrectomy, and avoiding unnecessary removal of the vitreous makes the procedure safer and saves time and cost to the facility. Alternatively, intravenous mannitol infusion can be given to dehydrate the vitreous humor by drawing out water which decreases vitreous mass and pressure. Unfortunately, this process is much slower than the direct fluid aspiration and thus less efficient.

Positive pressure in the vitreous cavity can also develop when large amounts of viscoelastic are placed in the sulcus. When placing a sulcus IOL or when performing endocyclophotocoagulation in glaucoma patients, a cohesive viscoelastic material is typically injected into the sulcus space. However, viscoelastic can track posteriorly through the zonules, leading to positive pressure in the posterior chamber. This shift leads to shallowing of the sulcus space, which can mistakenly lead the surgeon to inject additional viscoelastic into the sulcus space, further exacerbating the positive pressure. A 25-gauge or a 27-gauge needle on an open 3-mL syringe can be inserted 3.5mm posterior to the limbus in a pseudophakic eye through the pars plana. Left open to air for 10–15 seconds or with application of active aspiration, this syringe will allow for the egress of excess viscoelastic, thereby relieving the positive pressure in the posterior chamber.

## 4 Conclusions

In summary, aspiration of retrolental fluid with a short 25-gauge needle on a medium-size syringe is a simple and inexpensive method for immediate resolution of high IOP and shallow anterior chamber in cases of acute intraoperative fluid misdirection.

## Patient consent

Written consent to publish this case has not been obtained. This report does not contain any personal identifying information.

## Financial support

No financial support was received for the work being submitted, and none of the conflicts of interest listed are relevant to this work.

## Authorship

All authors attest that they meet the current ICMJE criteria for Authorship.

## Declaration of competing interest

NZG reports grant support from Biogen and Applied Genetic Technologies Corporation, consultation honoraria from Bionic Vision Technologies, and travel expenses from Medical Conference Planners International. SS reports consultant fees from Sight Sciences, Ivantis, Lumata Health, and Abbvie as well as equity in Lumata Health and sponsorship from Heidelberg Engineering. The following authors have no finandical disclosures: PS and RZ.
